# Bioinformatic Exploration of Hub Genes and Potential Therapeutic Drugs for Endothelial Dysfunction in Hypoxic Pulmonary Hypertension

**DOI:** 10.1155/2022/3677532

**Published:** 2022-11-28

**Authors:** Ai Chen, Weibin Wu, Siming Lin, Liangdi Xie

**Affiliations:** ^1^Department of Geriatrics, The First Affiliated Hospital of Fujian Medical University, Fuzhou, China; ^2^Fujian Hypertension Research Institute, The First Affiliated Hospital of Fujian Medical University, Fuzhou, China; ^3^Branch of National Clinical Research Center for Aging and Medicine, Fujian Province, Fuzhou, China; ^4^Fujian Provincial Clinical Research Center for Geriatric Hypertension Disease, Fuzhou, China; ^5^National Clinical Research Center for Aging and Medicine, Huashan Hospital, Fudan University, Fuzhou, China

## Abstract

Hypoxic pulmonary hypertension (HPH) is a fatal chronic pulmonary circulatory disease, characterized by hypoxic pulmonary vascular constriction and remodeling. Studies performed to date have confirmed that endothelial dysfunction plays crucial roles in HPH, while the underlying mechanisms have not been fully revealed. The microarray dataset GSE11341 was downloaded from the Gene Expression Omnibus (GEO) database to identify differentially expressed genes (DEGs) between hypoxic and normoxic microvascular endothelial cell, followed by Gene Ontology (GO) annotation/Kyoto Encyclopedia of Genes and Genomes (KEGG), Gene Set Enrichment Analysis (GSEA) pathway enrichment analysis, and protein-protein interaction (PPI) network construction. Next, GSE160255 and RT-qPCR were used to validate hub genes. Meanwhile, GO/KEGG and GSEA were performed for each hub gene to uncover the potential mechanism. A nomogram based on hub genes was established. Furthermore, mRNA-miRNA network was predicted by miRNet, and the Connectivity Map (CMAP) database was in use to identify similarly acting therapeutic candidates. A total of 148 DEGs were screened in GSE11341, and three hub genes (*VEGFA*, *CDC25A*, and *LOX*) were determined and validated via GSE160255 and RT-qPCR. Abnormalities in the pathway of vascular smooth muscle contraction, lysosome, and glycolysis might play important roles in HPH pathogenesis. The hub gene-miRNA network showed that hsa-mir-24-3p, hsa-mir-124-3p, hsa-mir-195-5p, hsa-mir-146a-5p, hsa-mir-155-5p, and hsa-mir-23b-3p were associated with HPH. And on the basis of the identified hub genes, a practical nomogram is developed. To repurpose known and therapeutic drugs, three candidate compounds (procaterol, avanafil, and lestaurtinib) with a high level of confidence were obtained from the CMAP database. Taken together, the identification of these three hub genes, enrichment pathways, and potential therapeutic drugs might have important clinical implications for HPH diagnosis and treatment.

## 1. Introduction

Hypoxic pulmonary hypertension (HPH) is a potentially fatal chronic pulmonary circulatory disease caused by hypoxia, primarily characterized by hypoxic pulmonary vasoconstriction and pulmonary vascular remodeling with poor prognostication. In recent years, the involvement of endothelial dysfunction in HPH has gradually attracted attention, and hypoxia-induced endothelial injury is considered as the initiating link of pulmonary artery endothelial dysfunction. Chronic hypoxia damages the integrity of the pulmonary artery intima, disturbs the endothelial barrier function, stimulates the abnormal proliferation of pulmonary artery smooth muscle cells (PASMC), and induces excessive deposition of extracellular matrix (ECM), finally, resulting in pulmonary artery medium and adventitia thickening [1]. Hypoxia also promotes the secretion of vasoconstrictor factors (such as endothelin-1 and 5-hydroxytryptamine) and decreases the secretion of relaxation factors (such as nitric oxide, prostacyclin, and atrial natriuretic peptide) in endothelial cells, thereby aggravating the vasoconstriction of pulmonary artery, leading to vascular remodeling [2, 3]. In addition, chronic hypoxia directs excessive proliferation of pulmonary artery endothelial cells (PAEC), intimal thickening, and lumen stenosis, all of which contribute to an increase in pulmonary arterial pressure (PAP). However, elevated PAP in turn induces the release of numerous cytokines, establishing a vicious loop that exacerbates the onset and progression of HPH. Therefore, it is anticipated that the discovery of efficient therapeutic medications targeting HPH would postpone or even reverse the advancement of HPH.

Unfortunately, there are currently no reliable biomarkers for the clinical diagnosis of HPH. Clinical drugs only regulate vascular function by upregulating the level of prostacyclins, promoting the expression of nitric oxide and cyclic guanosine monophosphate but only relieve symptoms and cannot comprehensively solve endothelial dysfunction from the root. Nevertheless, several studies have shown that many commercially available medicines for other illnesses have effective treatment benefits in HPH animal models [4–6]. In this paper, the Connectivity Map (CMAP) database is utilized to forecast several commercially available medications with strong therapeutic potential, and it is believed that the combination of current treatments could provide greater therapeutic benefits.

DNA microarrays represent a gene regulatory network between biology and computers, which make it possible to connect physiological cell states with gene expression patterns for studying underlying biological mechanisms and providing new opportunities for biomarker and unique potential drug discoveries and, finally, initiating gene therapy and prevention strategies [7–10]. Hub genes, highly functionally interconnected with other genes in candidate modules, usually play an essential role in pathophysiological function [11]. In this study, hub genes were defined as genes with high module membership (MM), measured by absolute value of Pearson's correlation (∣cor.weighted | >0.8), and high connectivity, which meant that the connectivity ranked at top 10% [12].

In this study, the gene expression profiles were obtained from the GEO database. The differential gene expression (DEGs) of microvascular endothelial cells from HPH was identified, and the related biological functions of DGEs regulated by hypoxia were analyzed by text mining, in order to deeply understand the biological molecular mechanisms of endothelial dysfunction in PHP.

## 2. Materials and Methods

### 2.1. Dataset Selection

**Figure 1:**
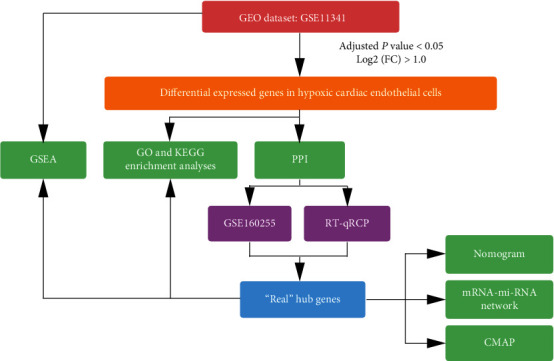
The study flow chart. GEO: Gene Expression Omnibus; GSEA: Gene Set Enrichment Analysis; GO: Gene Ontology; KEGG: Kyoto Encyclopedia of Genes and Genomes; PPI: protein-protein interaction; CMAP: Connectivity Map; RT-qPCR: real-time quantitative polymerase chain reaction.

The mRNA expression profile datasets GSE11341 [13] and GSE160255 [14] were downloaded from the GEO database (https://www.ncbi.nlm.nih.gov/geo/), which are located on the GPL96 [HG-U133A] Affymetrix Human Genome U133A Array and GPL23159 [Clariom_S_Human] Affymetrix Clariom S Assay, Human (includes Pico Assay), respectively. Human cardiac microvascular endothelial cells from GSE11341 were included to screening DEGs and hub genes, containing nine hypoxia samples (three for 3 h, 24 h, and 48 h, respectively) and three normoxia samples. Meanwhile, human pulmonary microvascular endothelial cells from GSE160255 were applied for validation of the target hub genes, in which three hypoxia and three normoxia sample were involved. The characteristics of the individual dataset were displayed in Supplementary Table 1, and the study flow chart design is shown in Figure 1.

### 2.2. DEG Analysis

The expression matrix of GSE11341 normalized by GEO2R (https://www.ncbi.nlm.nih.gov/geo/geo2r/) was represented by a box line plot. Reproducibility of the data was identified by principal component analysis (PCA). To select the DEGs, “limma” package of *R* software (version 3.6.3) [15] was utilized with cut-offs of adjusted *P* value (from the Benjamini-Hochberg method) <0.05 and log2 − absolute fold change (FC) > 1. Heat map was created using the “ComplexHeatmap” (version 2.2.0), and volcano map and box line plot were made by “ggplot2” (version 3.3.3) of *R* software (version 3.6.3).

### 2.3. Gene Ontology (GO) and Kyoto Encyclopedia of Genes and Genomes (KEGG) Pathway Enrichment of DEGs

In order to further understand biological functions of DEGs, GO and KEGG enrichment analyses were conducted with “clusterProfiler” (version 3.14.3) and visualized by “ggplot2” (version 3.3.3) [16]. The GO terms included 3 criteria: molecular function (MF), cellular component (CC), and biological process (BP).

### 2.4. Protein-Protein Interaction (PPI) Network Construction and Hub Gene Identification

The PPI networks of DEGs performed the Search Tool for the Retrieval of Interacting Genes (STRING; http://string.embl.de/), with a combined confidence score ≥ 0.9. CytoHubba, a plug-in of Cytoscape (version 3.7.2), was carried out to filter the top 10 hub genes of DEGs according to seven algorithms (stress, betweenness, radiality, closeness, EPC, degree, and MNC). Then, UpSet plot was visualized by UpSet*R* package (version 1.4.0).

### 2.5. Gene Set Enrichment Analysis (GSEA)

The goal of GSEA was to evaluate microarray data at the level of gene sets, which were selected with an absolute normalized enrichment score (∣NES∣) ≥ 1 and a false discovery rate (FDR) ≤ 0.25.

### 2.6. Development and Validation of Nomogram

Based on the identified hub genes obtained from GSE160255 and results of RT-qPCR, a nomogram was created to predict the prevalence of HPH in GSE11341. The calibration curves were applied to quantify the discrimination capacity of the nomogram-based prediction of HPH risk. Additionally, receiver operating characteristic (ROC) analysis was utilized to illustrate the discrimination efficiency and performance of the prediction model. Decision curve analysis (DCA) was performed to establish the clinical validity of the nomogram by calculating the net benefits at various threshold probabilities.

### 2.7. Potential Therapeutic Drugs Researching in CMAP

The CMAP (http://www.broad.mit.edu/cmap/) is a collection of genome-wide transcriptional expression data from cultured human cells treated with bioactive small molecules, which enables the discovery of decisive functional connections between drugs and genes based on similar and opposite gene expression profiles. Then, the upregulated DEGs were loaded, and 2D structures of candidate compounds were obtained from DrugBank.

### 2.8. mRNA-miRNA Regulatory Network Construction

The miRNet database [17] (https://www.mirnet.ca/) was applied to predict target miRNAs of the hub genes. Then, Cytoscape and UpSet plot were used to obtain the common miRNAs between seven algorithms (stress, eccentricity, radiality, closeness, EPC, degree, and MCC).

### 2.9. Human Umbilical Vein Endothelial Cell (HUVEC) Culture

HUVECs were purchased from the Shanghai Cell Bank, Chinese Academy of Science (Shanghai, China). Cells were grown in Dulbecco' s Modified Eagle' s Medium (DMEM; Thermo Fisher Scientific, USA) supplemented with 10% fetal bovine serum (FBS; Thermo Fisher Scientific, USA) at 37°C in a humidified atmosphere with 5% CO_2_ and divided into two groups: normoxia group and hypoxia group (1% O_2_/5% CO_2_/94% N_2_).

### 2.10. Real-Time Quantitative Polymerase Chain Reaction (RT-qPCR) of the Identified Hub Genes

Total RNA was extracted from all specimens using TRIzol Reagent (Vazyme Biotech, China) while RT-PCR was performed using the MMLV Reverse Transcriptase cDNA kit, according to the manufacturer's protocol. Amplification (Heruibio, HRF0032, China) was initiated with a 3 min predenaturation step at 93°C, followed by 40 cycles of 1 min at 93°C, 1 min at 55°C, and 1 min at 72°C, and then a final extension at 72°C for 5 min. SYBR-Green was used as a fluorophore, and qPCR was performed in triplicate using the Roche LightCycler96 System (Roche, Switzerland). The expression of hub genes was normalized to *GAPDH*, which was used as an internal control according to the 2^-*ΔΔ*Cq^ method. Each sample was run in triplicate and in three independent experiments. All primer sequences used in this study were as follows (Table 1).

### 2.11. Statistical Analysis

GraphPad Prism 7.0 (GraphPad Software, CA, USA) was used to analyze comparisons with unpaired Student's *t*-test for two groups, and one-way analysis of variance (ANOVA) for multiple groups and data was expressed as mean ± standard deviation. All experimental procedures were repeated in triplicate independently. *P* value <0.05 was considered statistically significant.

## 3. Results

### 3.1. Identification of DEGs

**Figure 2:**
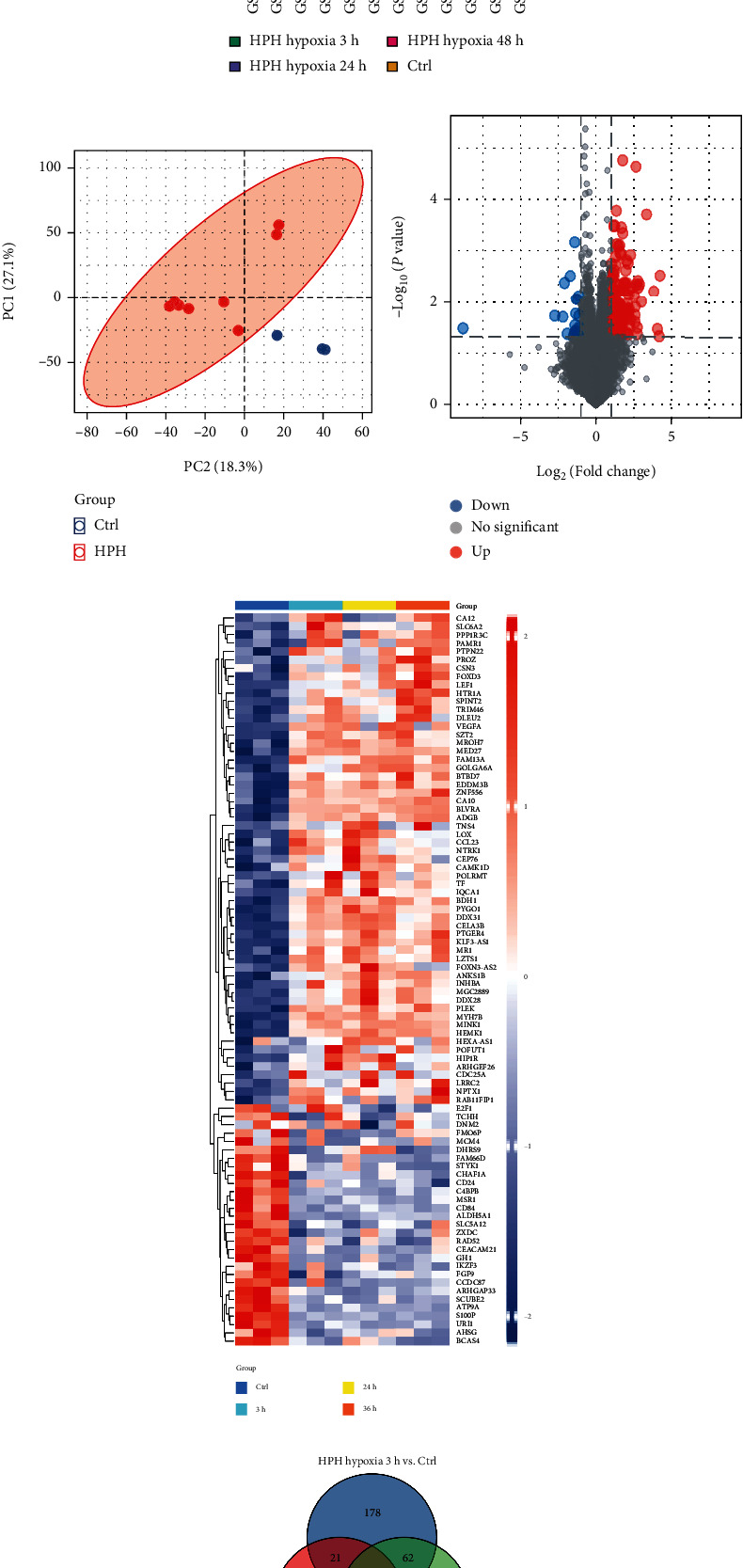
The results of DEG identification. (a) Box plot distribution. Horizontal line in the box = median of the statistic. (b) PCA after batch effect removement. (c) Volcano plot of all genes. (d) Heat map of DEGs screened by “limma” package. (e) Venn diagram showing overlap of DEGs in hypoxia 3 h vs. Ctrl, hypoxia 24 h vs. Ctrl, and hypoxia 48 h vs. Ctrl. PCA: principal component analysis; DEGs: differentially expression genes; Ctrl: control.

After data normalization, normalized gene expression profile was used for DEG analysis via GEO2R tool. The distribution trend of box plot was basically straight line (Figure 2(a)), and PCA showed good repeatability of the data (Figure 2(b)). 148 DEGs (125 upregulated and 23 downregulated) were screened according to the threshold of an adjusted ∣log2(FC) | >1 and *P* < 0.05. The volcano plot and heat map demonstrated the expression of DEGs between hypoxia groups (hypoxia) and control groups (Ctrl) (Figures 2(c) and 2(d)). And Venn diagram was applied to display the DEGs overlapped between the hypoxia 3 h vs. Ctrl, hypoxia 24 h vs. Ctrl, and hypoxia 48 h vs. Ctrl comparisons, which revealed 20 DEGs overlapped in these three groups (Figure 2(e)).

### 3.2. Functional Enrichment Analysis of DEGs

**Figure 3:**
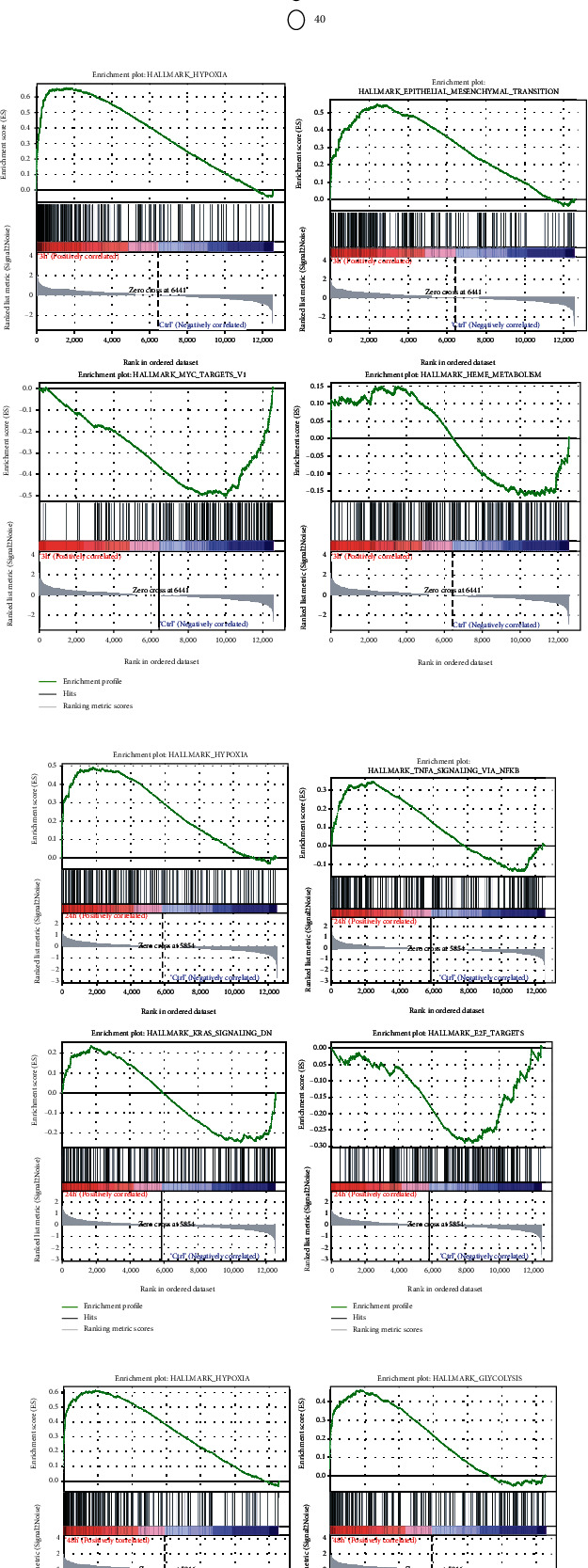
The results of GO/KEGG pathway enrichment and GSEA analyses. (a) GO/KEGG terms for DEGs. (b) Signaling pathways where the genes are predominant in hypoxia 3 h and Ctrl samples. (c) Signaling pathways where the genes are predominant in hypoxia 24 h and Ctrl samples. (d) Signaling pathways where the genes are predominant in hypoxia 48 h and Ctrl samples. GO: Gene Ontology; KEGG: Kyoto Encyclopedia of Genes and Genomes; GSEA: Gene Set Enrichment Analysis; DEGs: differentially expression genes; Ctrl: control.

As displayed in Figure 3(a), BP terms showed that the DEGs were enriched in “actin filament organization,” “ameboidal-type cell migration,” and “cell-substrate adhesion,” indicating that cell migration and adhesion might play a vital role in HPH. In terms of CC, “actin cytoskeleton,” “extracellular matrix,” and “collagen-containing extracellular matrix” were significantly enriched, suggesting that DEGs mainly had specific involvements in extracellular matrix. The major enriched MF terms of the DEGs were “actin binding,” “extracellular matrix structural constituent,” and “actin filament binding,” implying that DEGs enabled the matrix to resist compressive forces. Furthermore, “PI3K-Akt signaling pathway,” “focal adhesion,” and “proteoglycans in cancer” were obviously enriched in DEGs. In summary, the majority of these terms were involved in proliferation, migration, adhesion, and cancer, all of which played pivotal roles for endothelial dysfunction in HPH.

To further reveal the potential function of DEGs, GSEA was performed in hypoxia 3 h vs. Ctrl, hypoxia 24 h vs. Ctrl, and hypoxia 48 h vs. Ctrl. As shown in Figures 3(b)–3(d), enrichment of “HALLMARK_HYPOXIA” was observed in all hypoxia groups. In addition, “HALLMARK_EPIYHELIAL_MESENCHYMAL_TRANSITION” enriched in the hypoxia 3 h group, “HALLMARK_TNFA_SIGNALING_VIA_NFKB” enriched in the hypoxia 24 h group, and “HALLMARK_GLYCOLYSIS” enriched in the hypoxia 48 h group were shown, hinting that hypoxia-related high expression genes were associated with inflammation pathway, epithelial mesenchymal transition (EMT), and glycolysis pathway.

### 3.3. PPI Network Analysis and Hub Gene Recognition

**Figure 4:**
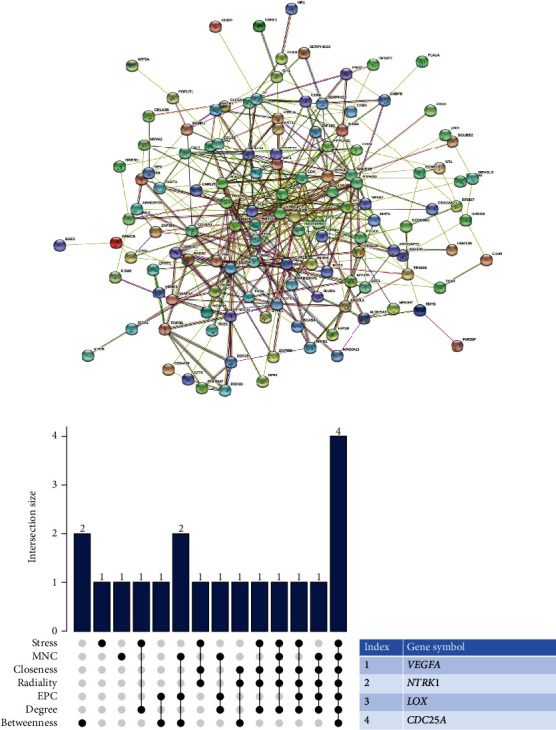
PPI network construction and hub genes analyses. (a) PPI network presented by STRING. (b) UpSet plot of results of CytoHubba. (c) Results of hub genes. PPI: protein-protein interaction. VEGFA: vascular endothelial growth factor A; NTRK1: neurotrophic receptor tyrosine kinase 1; LOX: lysyl oxidase; CDC25A: cell division cycle 25A.

A PPI network was constructed to find out the hub genes, including 128 nodes and 432 edges created by STRING (Figure 4(a)). Next, intersection of seven algorithms of CytoHubba was utilized to calculate the most credible result of the degree of DEGs (stress, betweenness, radiality, closeness, EPC, degree, and MNC) (Supplementary Table 2, Supplementary Figure 1), and finally, four hub genes were selected: *VEGF*A, *NTRK1*, *LOX,* and *CDC25A* (Figures 4(b) and 4(c)).

### 3.4. Validation of Hub Genes

**Figure 5:**
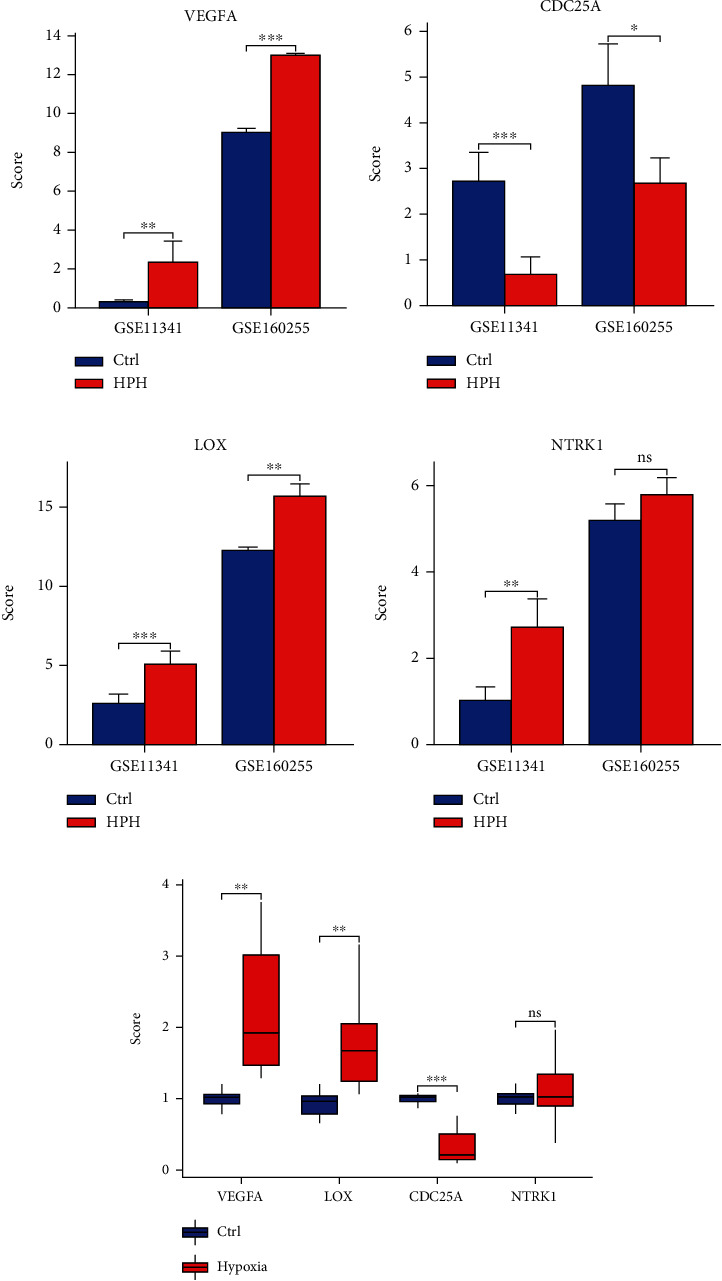
Validation of hub genes in GSE11341, GSE160255, and RT-qPCR. (a–d) Comparison of the expression of hub genes between GSE11341 and GSE160255. (a) VEGA, (b) CDC25A, (c) LOX, (d) NTRK1, and (e) the levels of identified hub genes in RT-qPCR. *N* = 6. Data are indicated as mean ± standard deviation. ^ns^*P* ≥ 0.05; ^∗^*P* < 0.05;^∗∗^*P* < 0.01;^∗∗∗^*P* < 0.001.

As shown in Figures 5(a)–5(d), results of GSE160255 were in partly accordance with our bioinformatic analysis. Obvious increment of *VEGF*A and *LOX* and reduction of *CDC25A* were observed in HPH groups (*P* < 0.05), while no significant difference of *NTRK1* was detected between HPH and Ctrl groups (*P* ≥ 0.05). And the four hub genes were labeled in the volcano plot that showed their expression levels (Supplementary Figure 2). According to the results of RT-qPCR, marked upregulation of *VEGFA* and *LOX* and downregulation of *CDC25A* in HUVECs under hypoxic exposure, but no significant change of the *NTRK1* expression was found (Figure 5(e)), were in agreement with that in GSE160255.

### 3.5. Functional Enrichment and GSEA to Reveal the Potential Functions of Hub Genes

**Figure 6:**
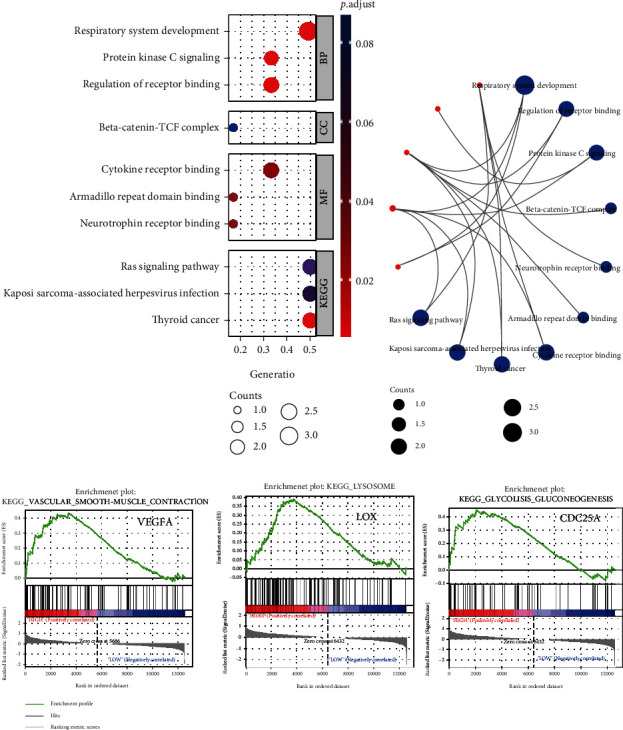
GO/KEGG/GSEA of identified hub genes. (a, b) GO/KEGG categories and pathways of hub genes. (c) Top KEGG pathways in the high-expression group of hub genes. GO: Gene Ontology; KEGG: Kyoto Encyclopedia of Genes and Genomes; GSEA: Gene Set Enrichment Analysis; VEGFA: vascular endothelial growth factor A; LOX: lysyl oxidase; CDC25A: cell division cycle 25A.

GO/KEGG enrichment analyses and GSEA were performed on three “real” hub genes (*VEGFA*, *CDC25A*, and *LOX*). As presented in Figures 6(a) and 6(b), in terms of BP, “respiratory system development,” “protein kinase C signaling,” and “regulation of receptor binding” were enriched, and “beta-catenin-TCF complexes” was enriched in terms of CC. Meanwhile, the major enriched MF terms of the hub genes were “cytokine receptor binding,” “armadillo repeat domain binding,” and “neurotrophin receptor binding.” Moreover, in the KEGG enrichment analysis, hub genes were particularly involved in “Ras signaling pathway,” “Kaposi sarcoma-associated herpevirus infection,” and “thyroid cancer.”

HPH samples from GSE11341 were divided into two groups according to the median of hub gene expression and subsequently conducted GSEA (Figure 6(c)). *CDC25A*-related groups were enriched in the “KEGG_GLYCOLYSIS_GLUCONENOGENESIS” pathway. Meanwhile, “KEGG_LYSOSOME” pathway was enriched in *LOX*-related groups, respectively, whereas “KEGG_VASCULAR_SMOOTH_MUSCLE_CONTRACTION” was enriched in *VEGF*A-related groups.

### 3.6. Construction and Evaluation of Nomogram Based on Identified Hub Genes

**Figure 7:**
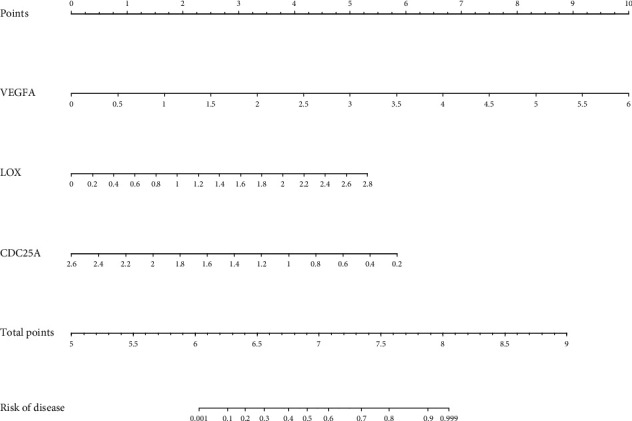
Construction of the nomogram of 3 identified hub genes. VEGFA: vascular endothelial growth factor A; LOX: lysyl oxidase; CDC25A: cell division cycle 25A.

**Figure 8:**
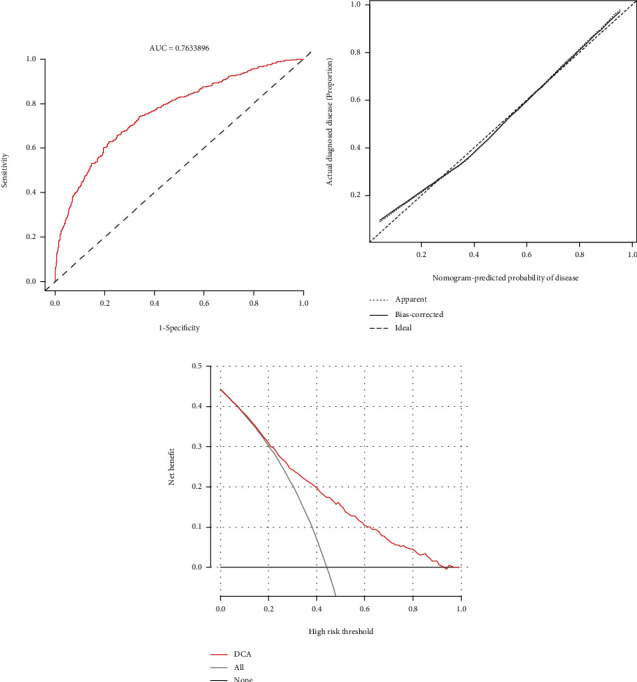
Evaluation of the nomogram. (a) ROC curve of the nomogram. (b) Calibration curve of the nomogram. (c) DCA of the nomogram. ROC: receiver operating characteristic; DCA: decision curve analysis.

A nomogram was constructed and presented in Figure 7 on the basis of above three hub genes (*VEGFA*, *CDC25A*, and *LOX*) for predicting HPH. Moreover, the ROC curve, calibration curve, and DCA were used to evaluate model performance. The ROC curve was displayed in Figure 8(a) (AUC: 0.763, 95% CI: 0.760-0.766), and the calibration curve of the nomogram demonstrated a good agreement between actual observation and prediction based on the nomogram (Figure 8(b)). The DCA shown in Figure 8(c) revealed that if the threshold probability was >14% and <92%, using the nomogram to estimate the incidence of HPH was more advantageous than the “treat none” or “treat all” strategy.

### 3.7. Construction of mRNA-miRNA Regulatory Network

**Figure 9:**
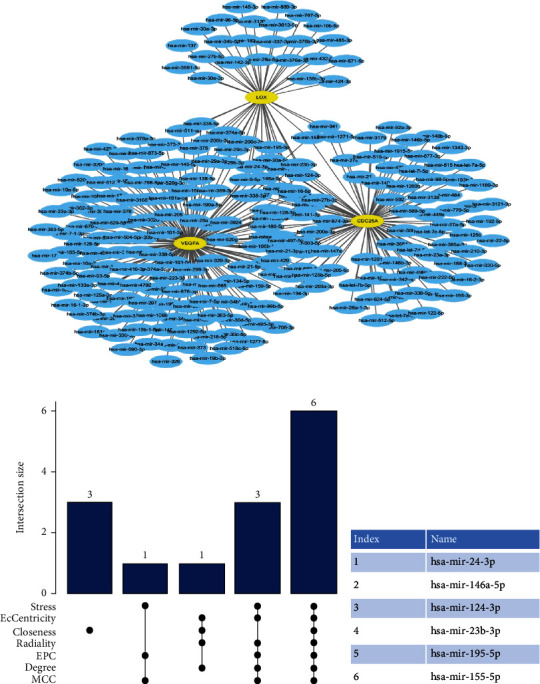
mRNA-miRNA regulatory network of identified hub genes. (a) mRNA-miRNA regulatory network presented by STRING. (b, c) UpSet plot of results of CytoHubba.

344 target miRNAs of hub genes were predicted via miRNA tool, and a coexpressed network of hub genes and miRNAs was established by Cytoscape which included 263 nodes and 344 edges (Figure 9(a)). Finally, six miRNAs (hsa-mir-24-3p, hsa-mir-124-3p, hsa-mir-195-5p, hsa-mir-146a-5p, hsa-mir-155-5p, and hsa-mir-23b-3p) were obtained by intersection of seven algorithms (Figures 9(b) and 9(c), Supplementary Figure 3).

### 3.8. Targeted Drug Prediction

DEGs were partitioned into up- or downregulated groups and then enriched with significantly changed genes obtained from treatment of small molecules from the CMAP database. As shown in Table 2, targeted compounds observed to induce more than 80% similar or opposite effects of HPH were selected (similar: levonorgestrel, naltrindole, and fluoxetine; opposite: procaterol, avanafil, and lestaurtinib). 2D structures of small molecule drugs as well as drug information were then obtained from the DrugBank (Supplementary Figure 4).

## 4. Discussion

In the present study, we filtered GSE11341, involving 12 samples from cardiac microvascular endothelial cells which were divided into four groups (HPH hypoxia 3, 24, 48 h, and normoxia), screening 148 DEGs to further explore the hub genes, key pathways, and potential drugs in response to hypoxia in HPH. Next, 148 DEGs were uploaded to the hub modules in the STRING database, selecting confidence > 0.9 to construct PPI network, where genes with a connectivity degree of ≥8 were also defined as common hub genes. The common hub genes both confirmed in GSE160255 and RT-qPCR were regarded as “real” hub genes for further analysis. Finally, three “real” hub genes and potential mechanisms of endothelial dysfunction in HPH were identified. Moreover, six significant upstream miRNAs were predicted by seven algorithms, and several drugs inducing similar or opposite effects response to hypoxia were found by CMAP.

The results of GO analysis suggested that the EDGs were markedly enriched in the migration and adhesion (actin filament organization, ameboidal-type cell migration, cell-substrate adhesion, actin filament binding, extracellular matrix structural constituent, and focal adhesion) and cancer-like response (proteoglycans in cancer), which was in accordance with pathogenesis mentioned before [18, 19]. Additionally, KEGG analysis indicated that PI3K-Akt signaling pathway was significantly enriched, which was consistent with the previous demonstration that hypoxia response involves crosstalk with proliferation and apoptosis mechanisms [20, 21]. And research conducted by Liu et al. demonstrated that the immune response, inflammatory response, cellular response to interleukins, cell cycle, and leukocyte migration were highly enriched, suggesting that immune and inflammatory response pathways participate in HPH [22], which was partially consistent with the hypoxia-related inflammatory response enriched in this study.

According to determination of synthesize seven algorithms, four hub genes (*VEGF*A, CDCD25A, *LOX*, and *NTRK1*) were identified. Among them, VEGFA (vascular endothelial growth factor A) is a well-known angiogenic factor induced by hypoxia [23, 24]. Abundant evidences [25, 26] suggest that VEGF-induced angiogenesis is one of several key hypoxia adaptations, and HIF-1*α*/VEGF signaling pathway is responsible for hypoxia-induced microvascular remodeling. CDC25A (cell division cycle 25 A) was considered as an essential mediator in the process of hypoxia-mediated cell cycle arrest, and Queiroz de Oliveira [27] recently demonstrated that CDC25A protein and mRNA levels decreased in human tumor cells under hypoxia conditions, proposing a new hypothesis that hypoxia-mediated reduction of CDC25A might confer protection from hypoxic conditions, contributing to cell survival. And the observation that LY294002, a PI3K/Akt inhibitor, reduced the expression of CDC25A, indicating involvement of PI3K/Akt signaling in the CDC25A expression [28]. Similar to our findings, in addition to its well-established involvement in cell cycle control, Liang et al. discovered that CDC25A plays a crucial part in the Warburg effect, recognized as the shift from oxidative phosphorylation to glycolysis in pulmonary hypertension (PH) [29]. However, there have not been numerous reports of CDC25A in endothelial cells. In this research, although downregulation of CDC25A was similarly observed in GSE11341, GSE160255, and results of RT-qPCR, whether hypoxia-mediated CDC25A degradation contributing to cell proliferation remained to be determined. LOX (lysyl oxidase) was identified as a hypoxia-responsive gene and found it to be regulated HIF-1, and hypoxia-induced LOX played a key role in tumor metastasis. Meanwhile, it has been demonstrated that LOX was also essential for stimulating endothelial cells and angiogenesis. However, LOX itself was not responsible for promoting angiogenesis but indirectly promoted angiogenesis by upregulating of VEGF [30]. In this study, we concluded that LOX might play important parts in angiogenesis triggered by hypoxia. Neurotrophic tropomyosin-receptor kinase 1 (NTRK1) is a nerve growth factor (NGF) receptor and actively involved in developing, protecting, and maintaining neurons. And the three alternative splicings of NTRK1 (TrkAI, TrkAII, and TrkAIII) have been described. Studies have shown that increased expressions of regular NTRK1 isoforms (TrkAI/II) in normoxia played an antioncogenic role, while under hypoxic conditions, the upregulation of TrkAIII plays an oncogenic role in tumor progression and metastasis PI3K/Akt signaling [31]. Nevertheless, NTRK1's function in endothelial cells remains unidentified. This study evaluated the levels of NTRK1 in endothelial cells from three different sources and indicated that hypoxia significantly affected NTRK1 levels in cardiac microvascular endothelial cells, but not in pulmonary vascular and HUVECs, which required further investigation.

To further explore the potential pathogenesis of the hub genes in HPH, GO/KEGG analysis and GSEA were performed. Combining these two enrichment analysis methods, more credible results were obtained. Multiple cell proliferation, receptor-associated protein activity, cancer-like growth, and carbohydrate metabolism-related pathways were enriched, implying that these hub genes might contribute to angiogenesis and glycolysis processes in HPH, which was in accordance with our previous study that vascular cells in MCT-induced PH take up energy similarly to tumor cells by producing energy through glycolysis rather than oxidative phosphorylation, in turn promoting cell proliferation [32]. Moreover, nomogram could quantify and forecast the likelihood of a clinical occurrence in order to aid clinical decision-making and risk stratification [33]. And in this research, a nomogram prediction model was developed based on the association between identified hub genes (*VEGFA*, *LOX* and *CDC25A)* and HPH, followed by the consistency analysis and discrimination evaluation, suggesting that the model had a reasonable ability to discriminate between high-risk and low-risk individuals, although clinical observation and imaging examination were still required for confirmation.

Moreover, potential drugs induce or reverse the altered expression of the DEGs through CMAP analysis. Among the drugs, procaterol is a well-known intermediate-acting *β*2 adrenoreceptor agonist, endothelial NO synthase (eNO) pathway is responsible for procaterol-mediated pulmonary arterial relaxation, and long-term administration might also continue to improve pulmonary hemodynamics [34]. Avanafil is a highly selective and potent oral phosphodiesterase type 5 inhibitor approved for erectile dysfunction, and FDA recently initiates discussions of expanding its indication to potentially include improvement in endothelial dysfunction [35, 36]. Compared with the traditional phosphodiesterase type 5 inhibitor, sildenafil, avanafil has the advantage of faster onset of action. It has a long half-life and low administration frequency and could rapidly dilate arteries within 15 to 20 minutes. Nevertheless, it is inevitable that all phosphodiesterase type 5 inhibitors have a similar flaw, namely, that they have no obvious improvement on right heart dysfunction and have specific restrictions in the treatment of advanced PH [37]. Therefore, a large number of clinical trials might be required to verify whether avanafil could be used as a first-in-class medication for HPH treatment. Lestaurtinib, a tyrosine kinase inhibitor, showed its proliferation suppression and pulmonary artery relaxation by targeting JAK/STAT signaling in PH [38]. Similar to lestaurtinib, imatinib is currently used in the treatment of chronic myeloid leukemia because of its inhibitory tyrosine kinase and blocking cell proliferation. Imatinib prevents the PH progression by limiting the activation of platelet-derived growth factor receptors, consequently reducing the creation of neovascularization intima and the migration of smooth muscle cells to the endodermis [39]. Imatinib (15 mg/kg) was found to reverse cardiopulmonary remodeling in animals, suggesting that low doses could partially reverse PH [4]. However, no trial has shown that lestaurtinib is superior than imatinib for the treatment of PH.

Inevitably, there are some limitations and shortfalls of bioinformatic analysis. Firstly, these analyses are all based on the GEO database other than sequencing results, which might reduce the reliability of our findings. Secondly, few databases on the detection of cardiac microvascular endothelial cells in HPH have been performed, so other sources of endothelial cells were used to confirm the results. Thirdly, current investigations have been limited by the lack of adequate experimental validation of identified hub genes. In the future, further gene functional verification experiments should be designed to validate the underlying mechanisms of action of these hub genes in hypoxia.

In conclusion, we successfully identified three “real” hub genes (*VEGF*A, *CDC25A*, and *LOX*), and six crucial miRNAs (hsa-mir-24-3p, hsa-mir-124-3p, hsa-mir-195-5p, hsa-mir-146a-5p, hsa-mir-155-5p, and hsa-mir-23b-3p) associated with endothelial dysfunction in HPH based on bioinformatic analysis. Abnormalities in the pathway of vascular smooth muscle contraction, lysosome, and glycolysis might play important roles in HPH pathogenesis. And on the basis of the identified hub genes, a practical nomogram is developed. Furthermore, three drugs, namely, procaterol, avanafil, and lestaurtinib, provided novel insights into the treatment of HPH. However, specific underlying experiments were required to validate the above results.

## Figures and Tables

**Table 1:** Primer sequences in this study.

Primer	Sequence
*VEGF*A-F	5′ ATCGAGTACATCTTCAAGCCAT 3′
*VEGF*A-R	5′ GTGAGGTTTGATCCGCATAATC 3′
*CDC25A*-F	5′ CAGTTGCTGGGAAACATCAGGA 3′
*CDC25A*-R	5′ ATGTGGCCTCCCTCGTATTC 3′
*LOX*-F	5′ GAGTCCTGGCTGTTATGATACCTA 3′
*LOX*-R	5′ GATGTCCTGTGTAGCGAATGTC 3′
*NTRK1*-F	5′ CACCAGAGGTCTACGCCATC 3′
*NTRK1*-R	5′ TCCAGGTAGACAGGAGGTGC 3′
*GAPDH*-F	5′ GGTGTGAACCATGAGAAGTATGA 3′
*GAPDH*-R	5′ GAGTCCTTCCACGATACCAAAG 3′

**Table 2:** Researching results in CMAP.

	CMAP name	Cell line	Score	Function	Targets
Hypoxia 3 h vs. Ctrl	Levonorgestrel	VCAP	0.9699	Ovulation, cervical mucus changes, and hormone therapy	Progesterone receptor, 3-oxo-5-alpha-steroid 4-dehydrogenase 1, and 4-estrogen receptor alpha
	Procaterol	NEU	-0.8991	Bronchodilator	Beta-2 adrenergic receptor
Hypoxia 24 h vs. Ctrl	Naltrindole	SKBR3	0.9009	Antagonist analog of opiate enkephalin	Delta opioid receptor
	Avanafil	HT29	-0.9231	Inhibition of cGMP-specific phosphodiesterase type 5 (PDE5)	cGMP-specific 3′,5′-cyclic phosphodiesterase
Hypoxia 48 h vs. Ctrl	Fluoxetine	HA1E	0.9193	Selective serotonin reuptake inhibitor (SSRI)	Sodium-dependent serotonin transporter
	Lestaurtinib	HCC515	-0.8995	Inhibition of FMS-like tyrosine kinase 3 (FLT3) autophosphorylation	FMS-like tyrosine kinase 3 (FLT3)

## Data Availability

The datasets generated and/or analyzed during the current study are available from the corresponding author on reasonable request.

## Supplementary Materials

Supplementary 1 Supplementary Figure 1: hub genes shown by CytoHubba from seven algorithms.

Supplementary 2 Supplementary Figure 2: the volcano plot of GSE160255. The four hub genes were labeled in the volcano plot which showed their expression levels.

Supplementary 3 Supplementary Figure 3: hub miRNA targeted identified hub genes shown by CytoHubba.

Supplementary 4 Supplementary Figure 4: 2D structure of the candidate compounds.

Supplementary 5 Supplementary Table 1: characteristics of the individual dataset.

Supplementary 6 Supplementary Table 2: the top 10 hub genes of 7 algorithms in CytoHubba.
