# Supplementary figures and images for: Bioinformatic Exploration of Hub Genes and Potential Therapeutic Drugs for Endothelial Dysfunction in Hypoxic Pulmonary Hypertension
Source: Comput Math Methods Med. 2022 Nov 28;2022:3677532. doi: 10.1155/2022/3677532 (PMC9723419; doi:10.1155/2022/3677532)

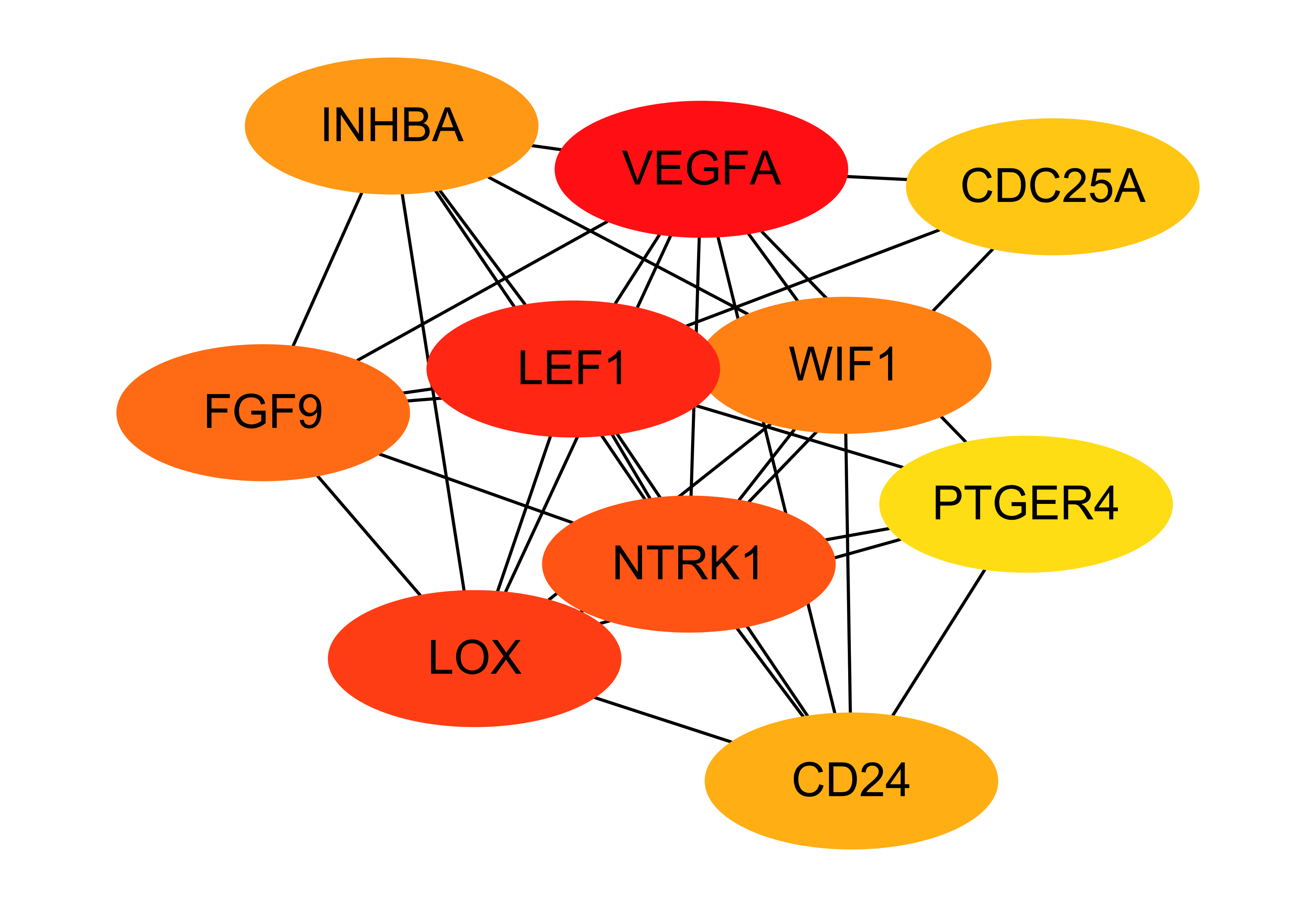


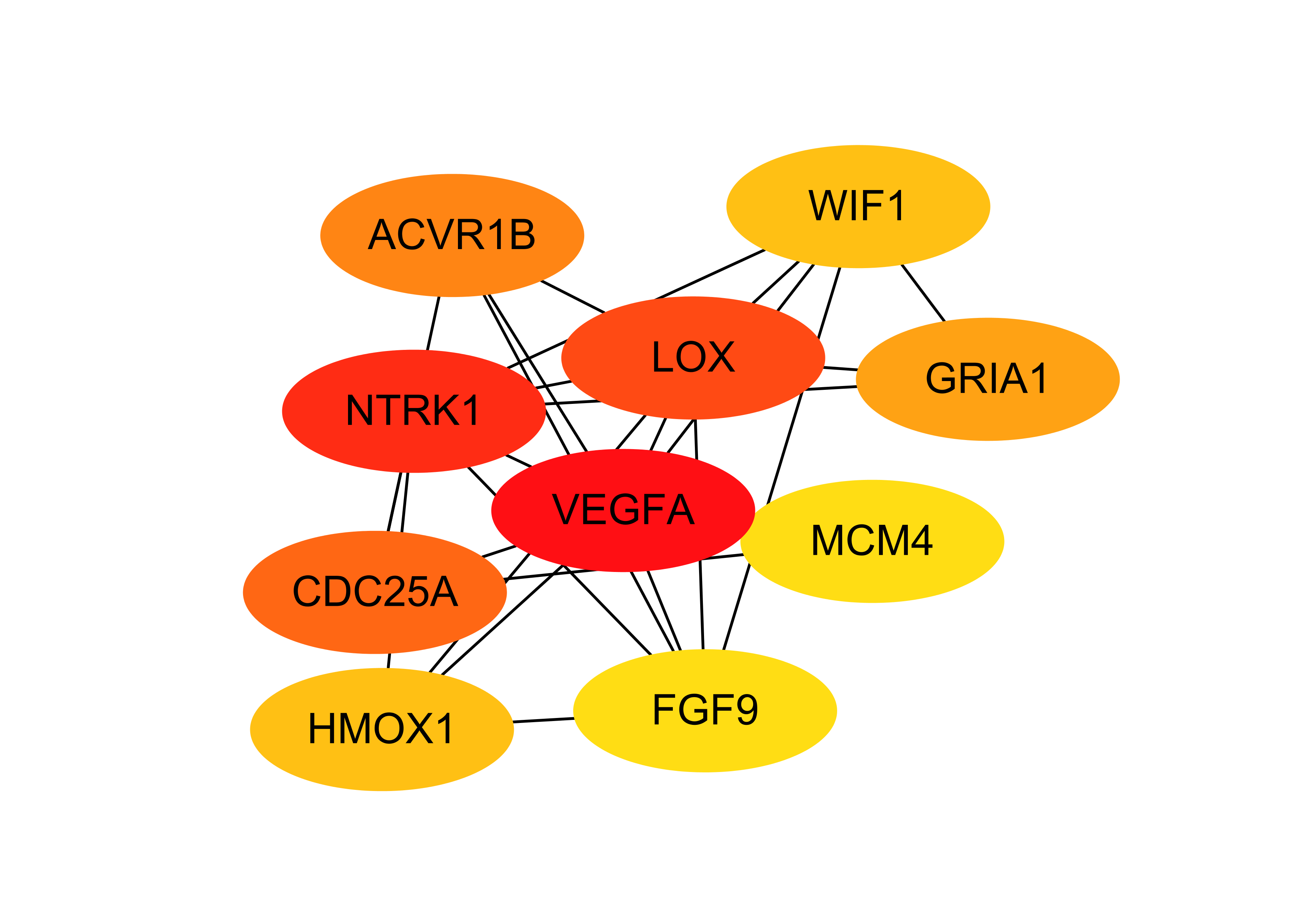


MNC

F

Closeness


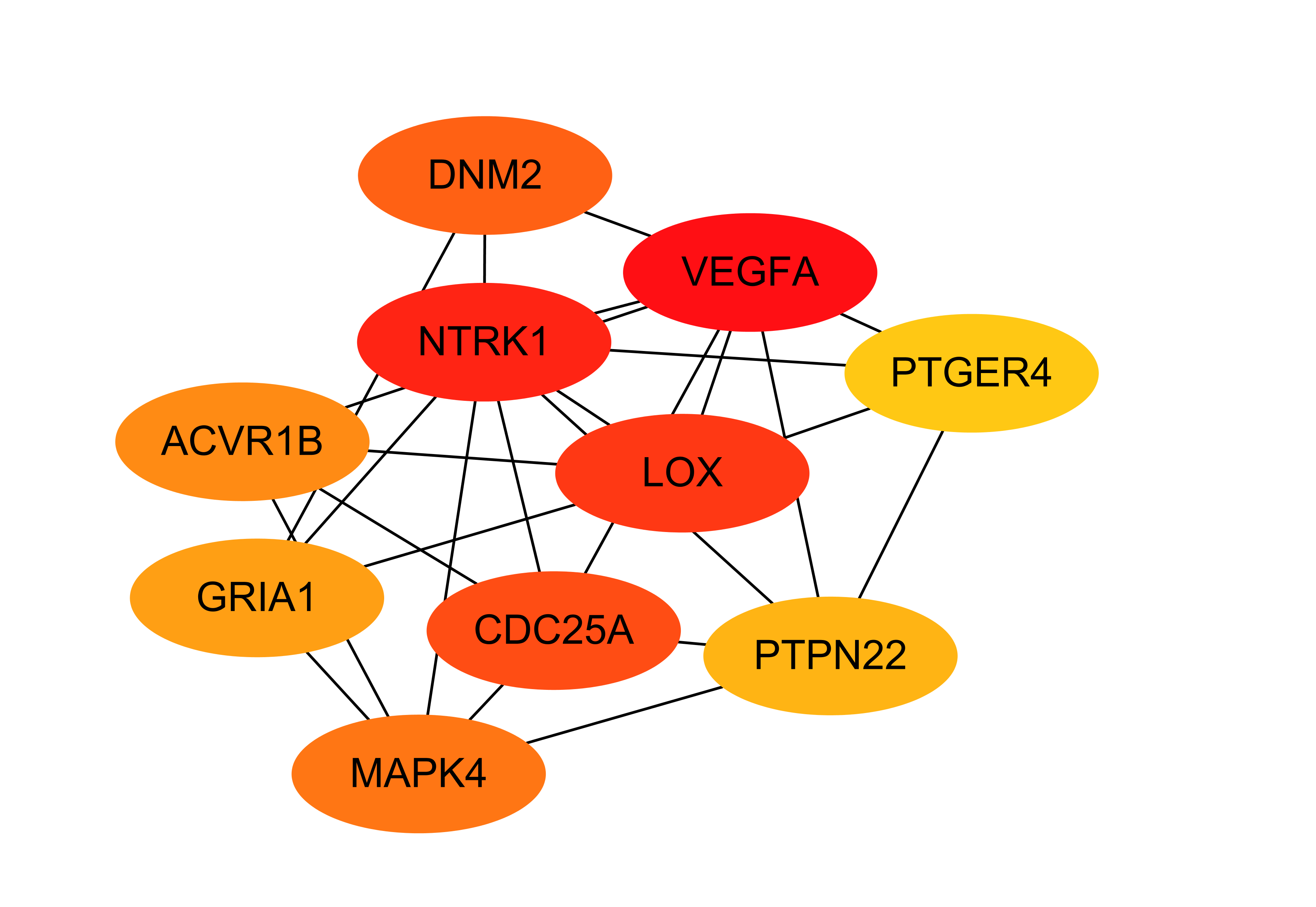


E

Radiality


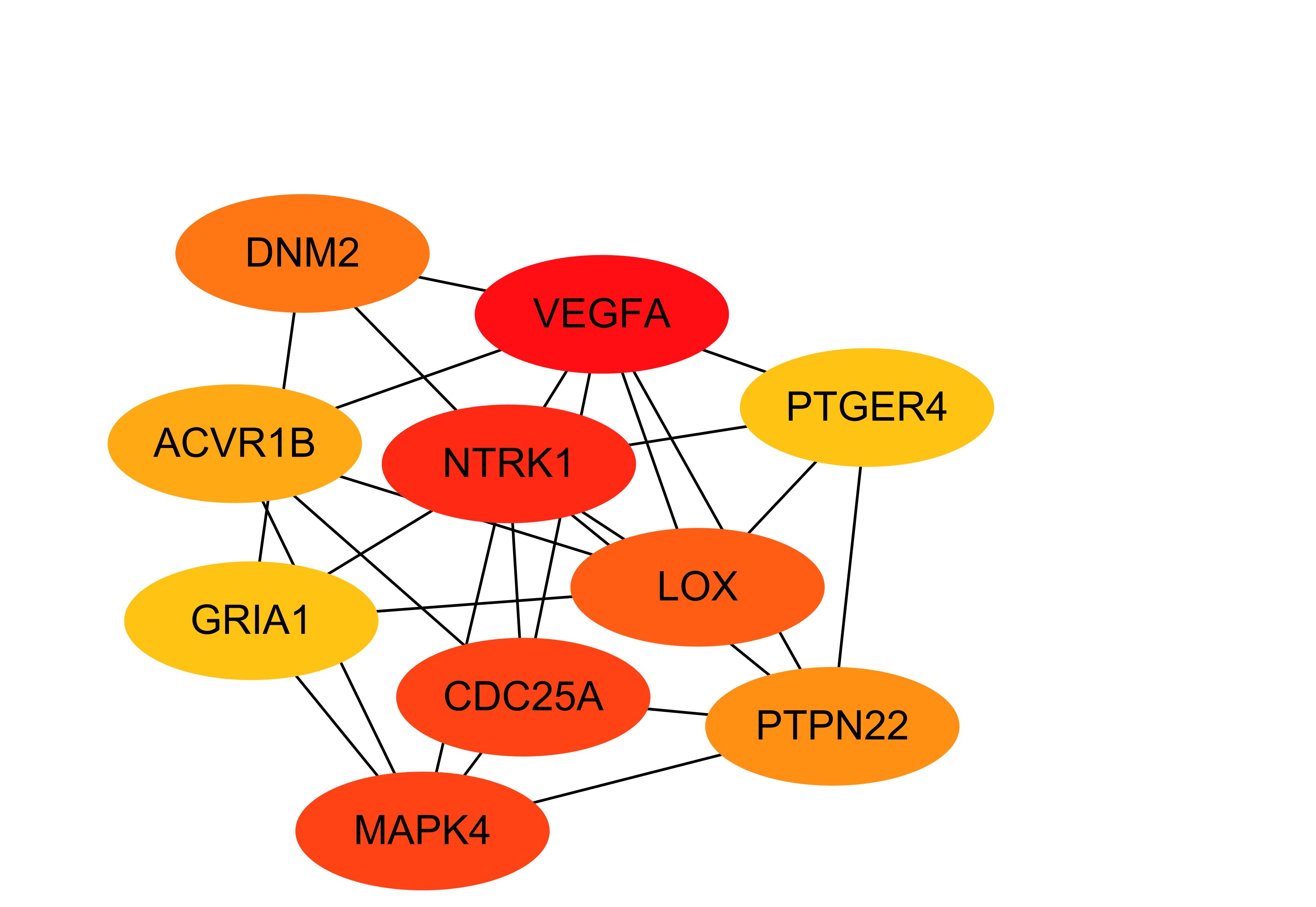


D


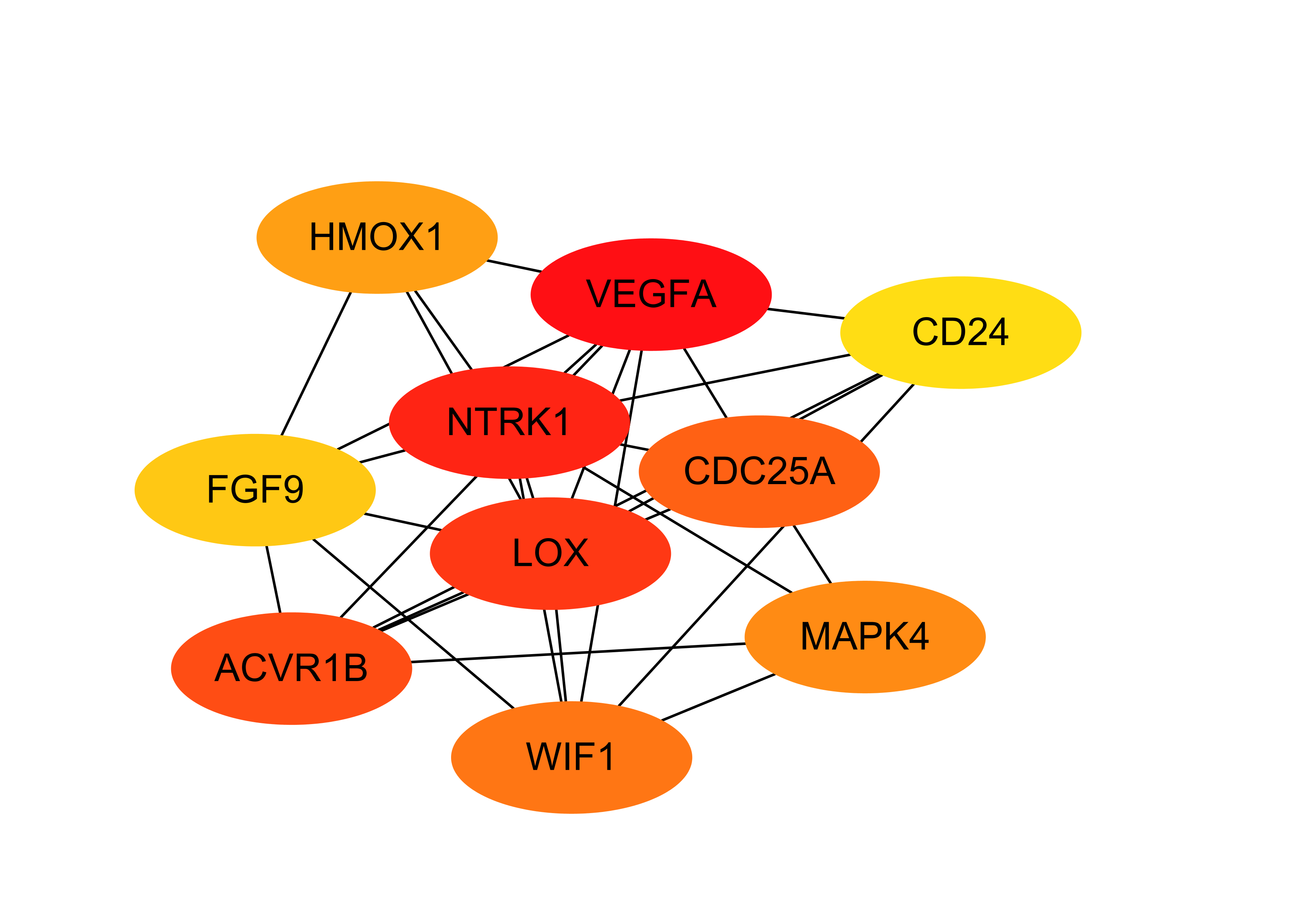


EPC

C


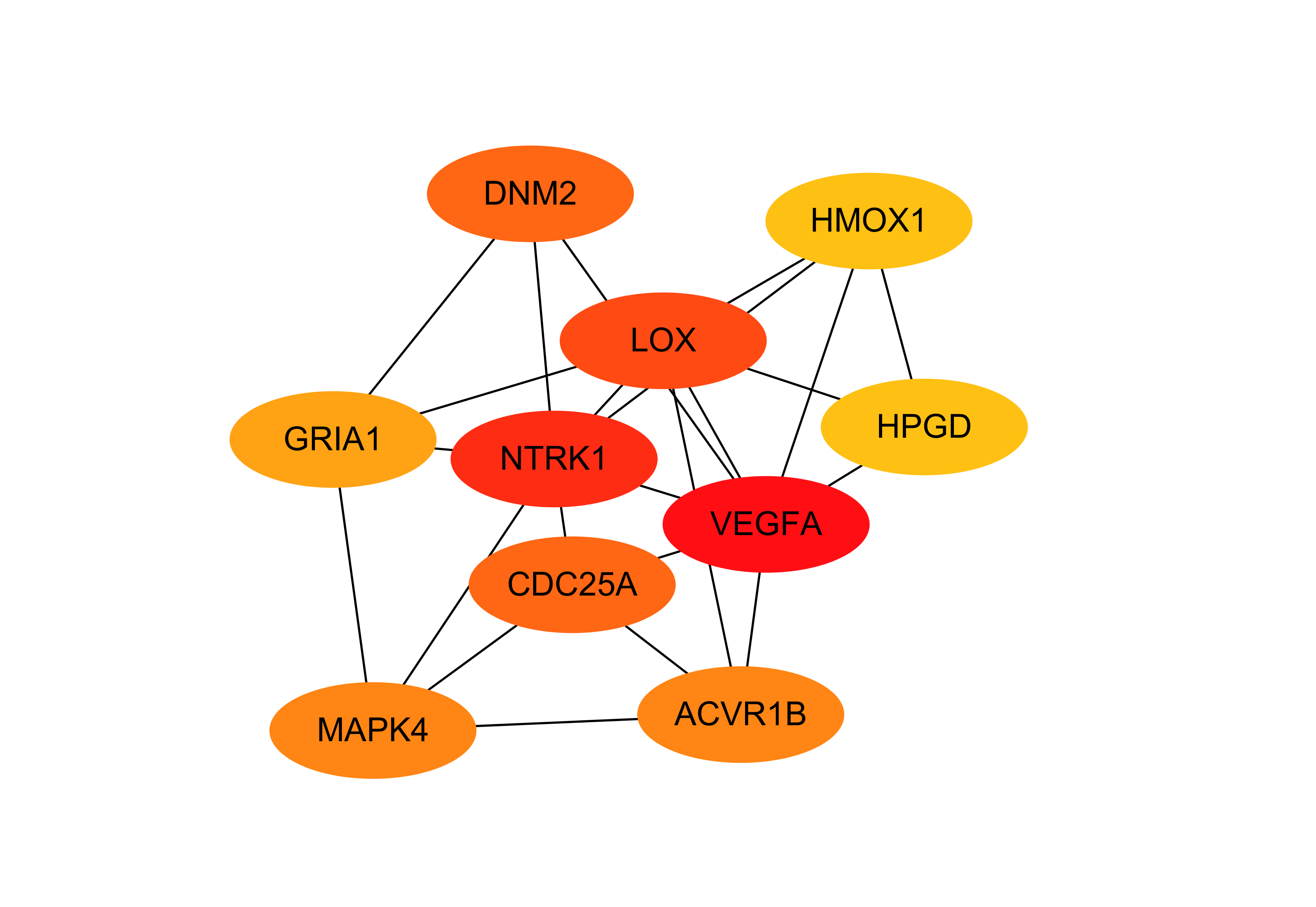


Degree

Betweeness

A

B

Stress


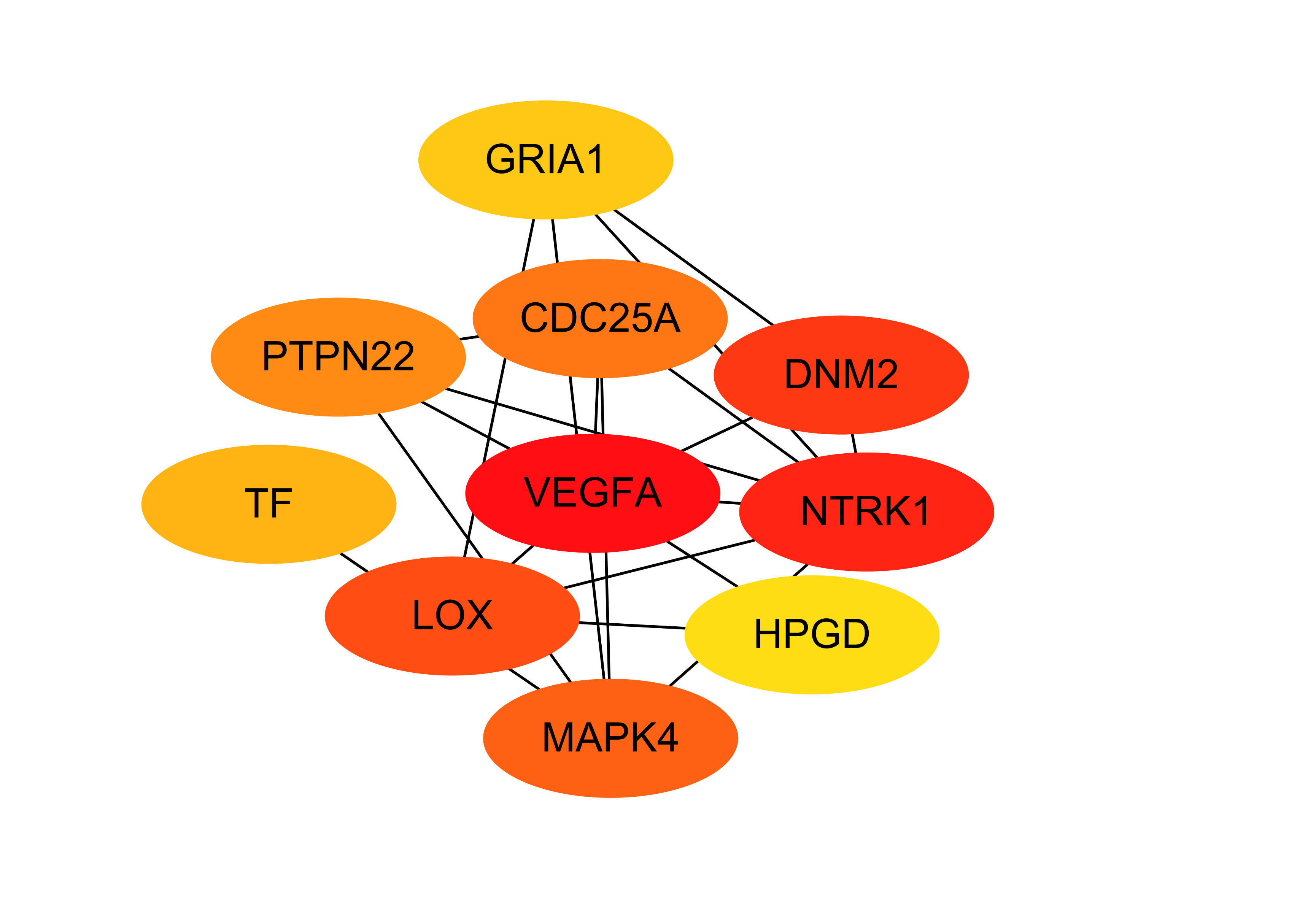


G

Supplement: Supplementary 1 — Supplementary Figure 1: hub genes shown by CytoHubba from seven algorithms. [file 3677532.f1.docx]

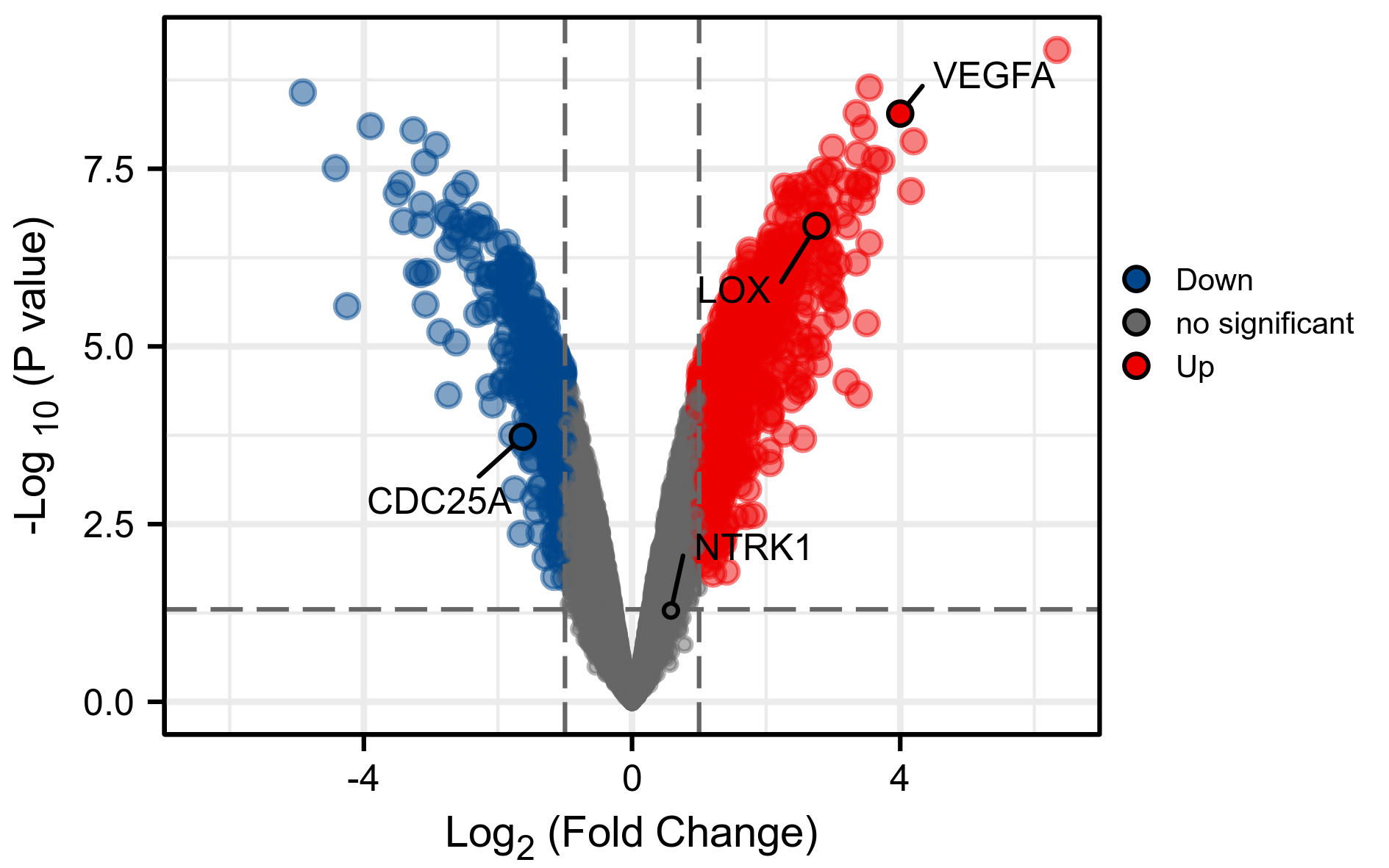

Supplement: Supplementary 2 — Supplementary Figure 2: the volcano plot of GSE160255. The four hub genes were labeled in the volcano plot which showed their expression levels. [file 3677532.f2.docx]

F

E

D

C

A

B

EPC


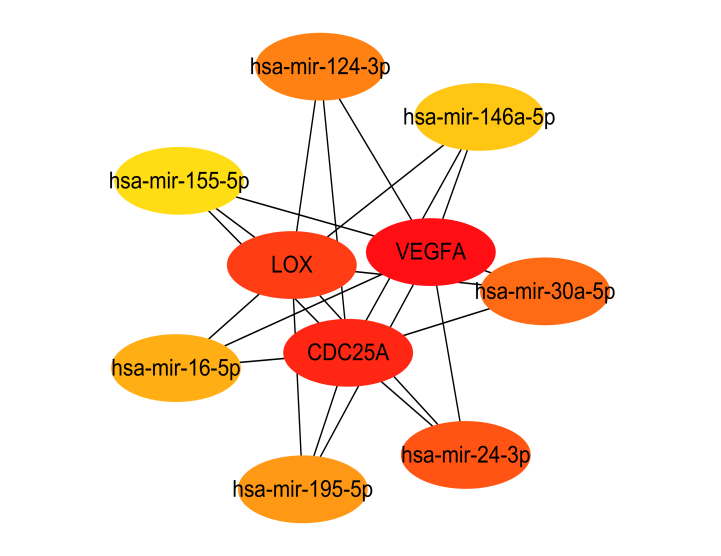


EcCentricity


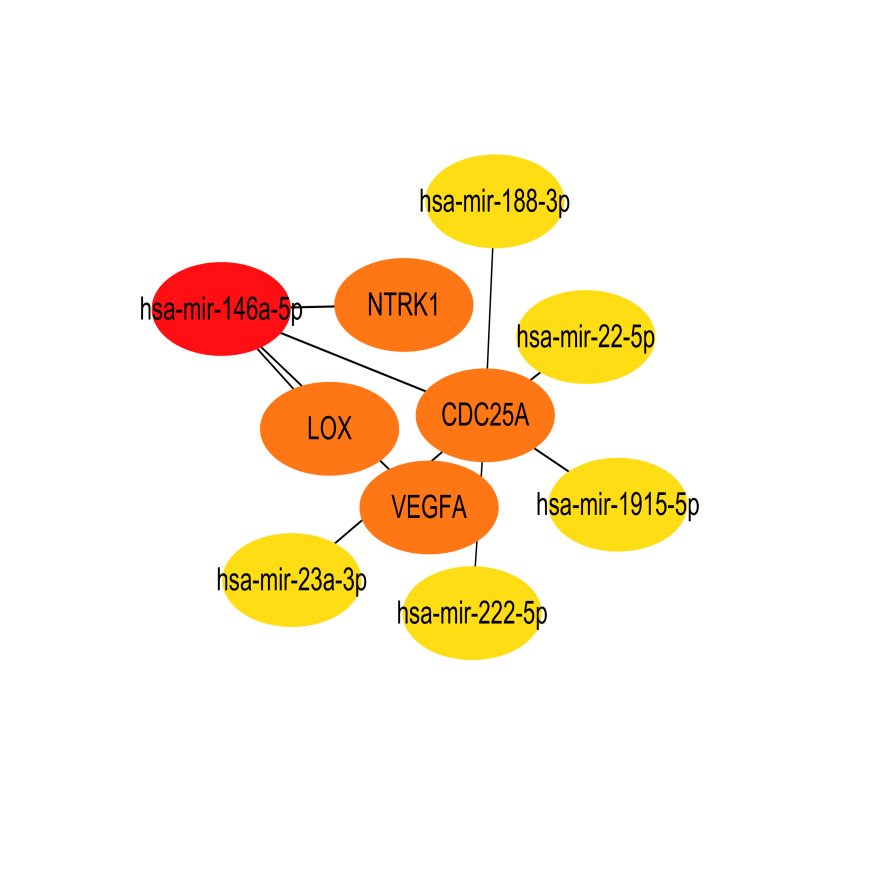


Degree


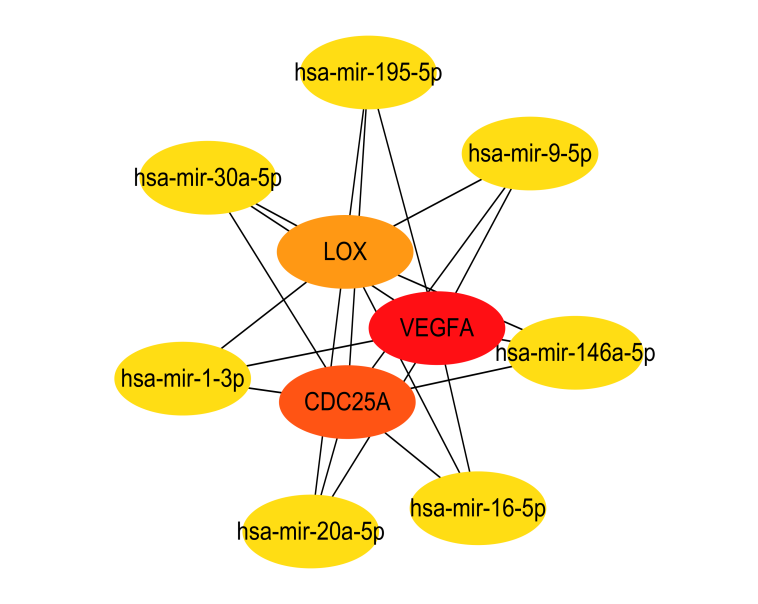


Radiality


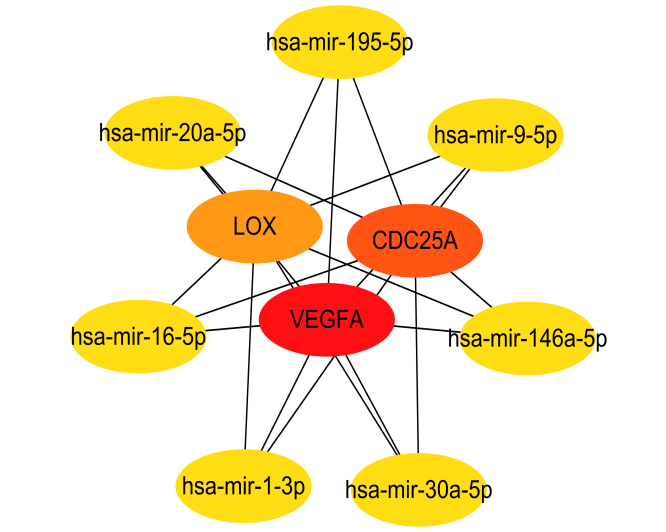


MCC


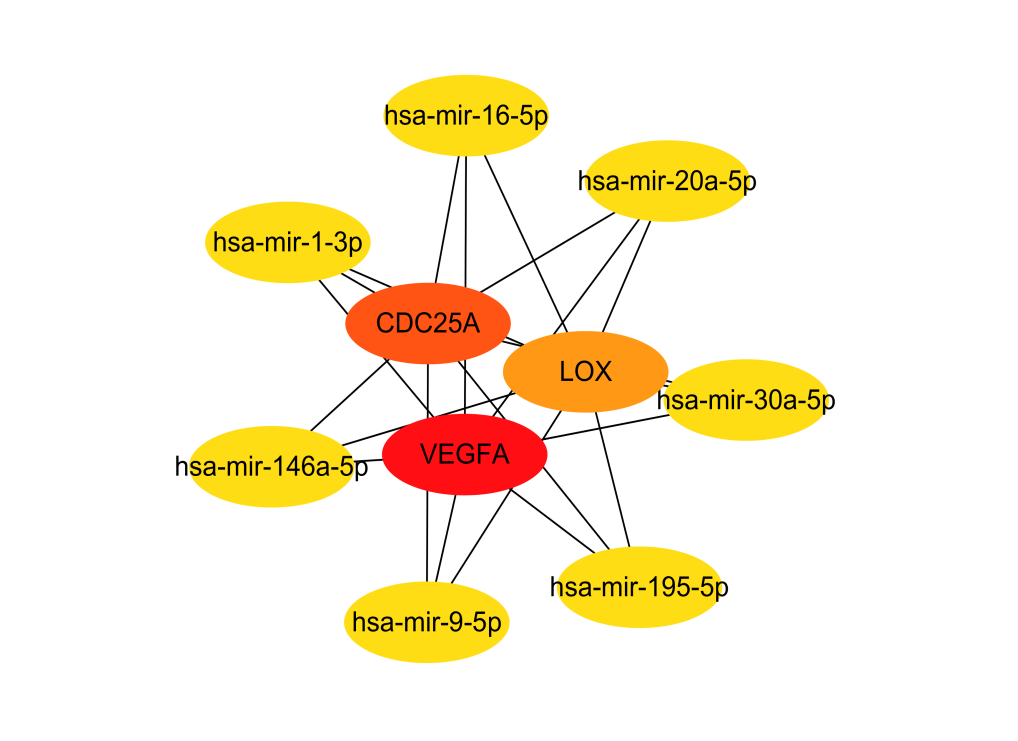


Closeness


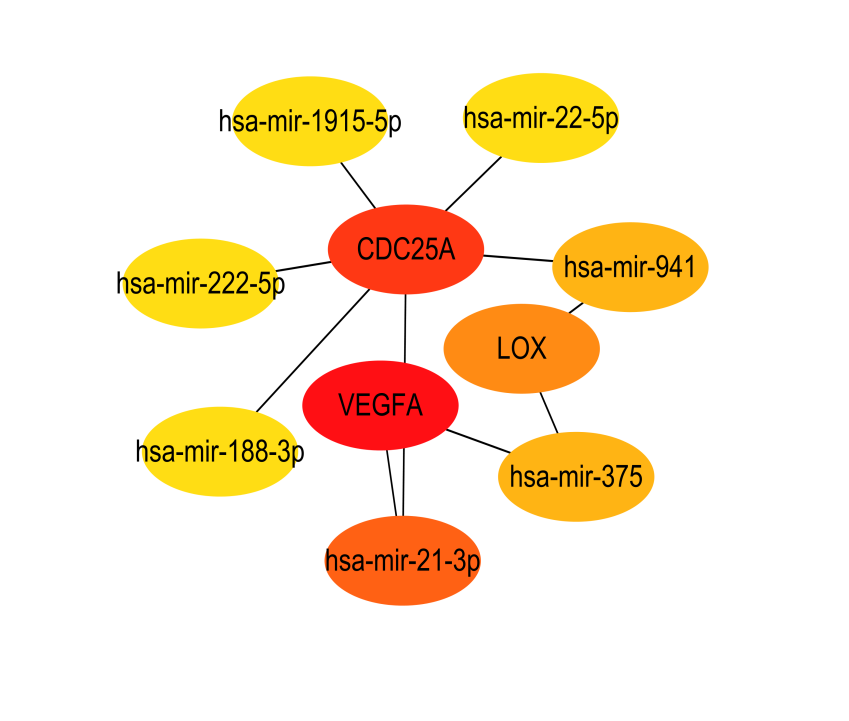


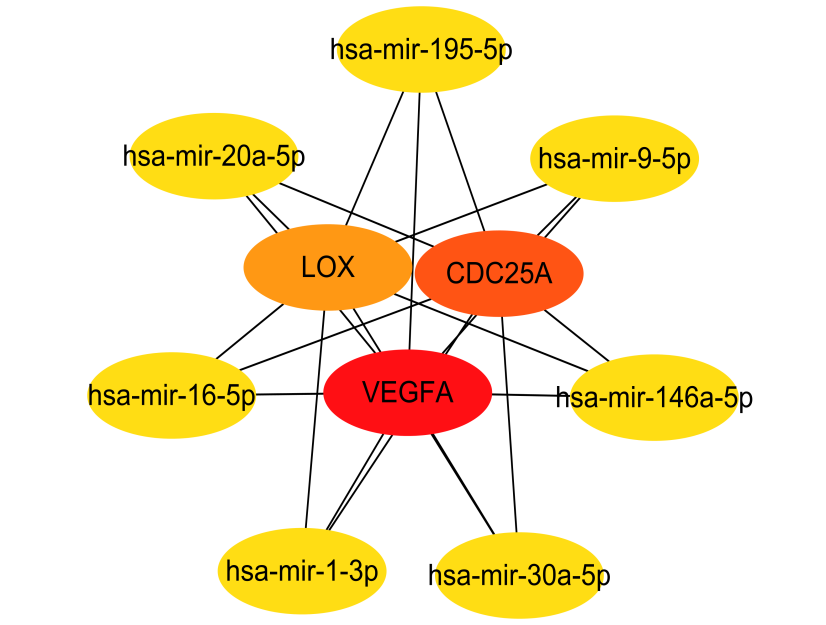


Stress

G

Supplement: Supplementary 3 — Supplementary Figure 3: hub miRNA targeted identified hub genes shown by CytoHubba. [file 3677532.f3.docx]

F

E

D

C

A

B


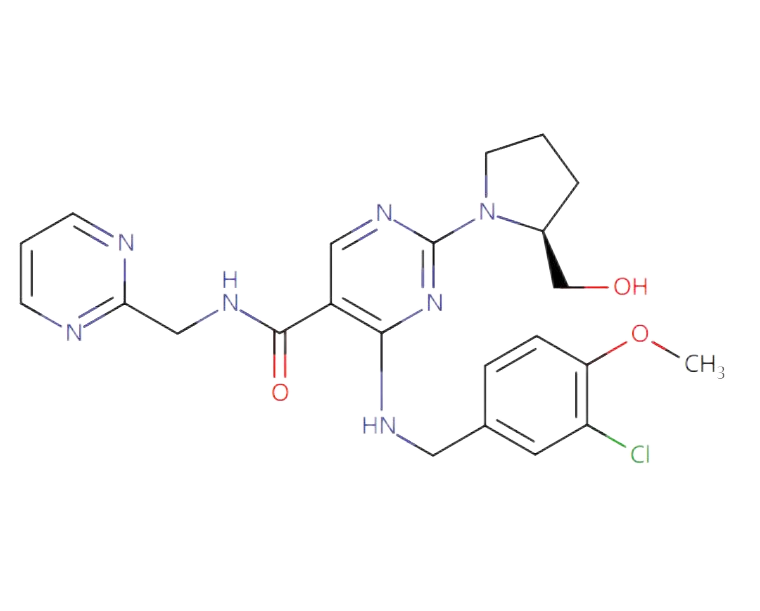


avanafil

procaterol


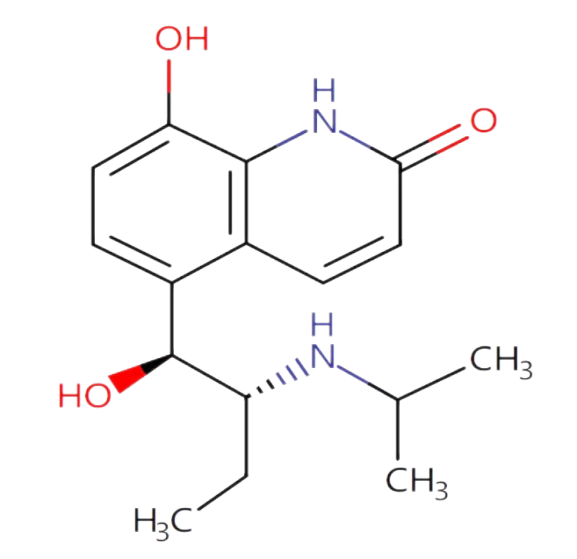


levonorgestrel

lestaurtinib


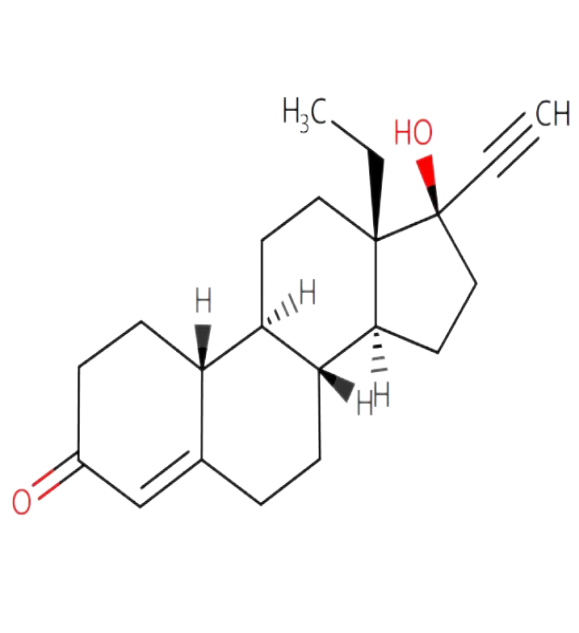


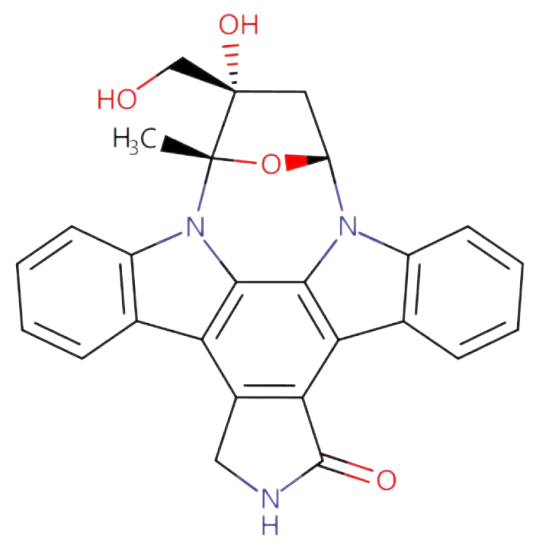


fluoxetine

naltrindole


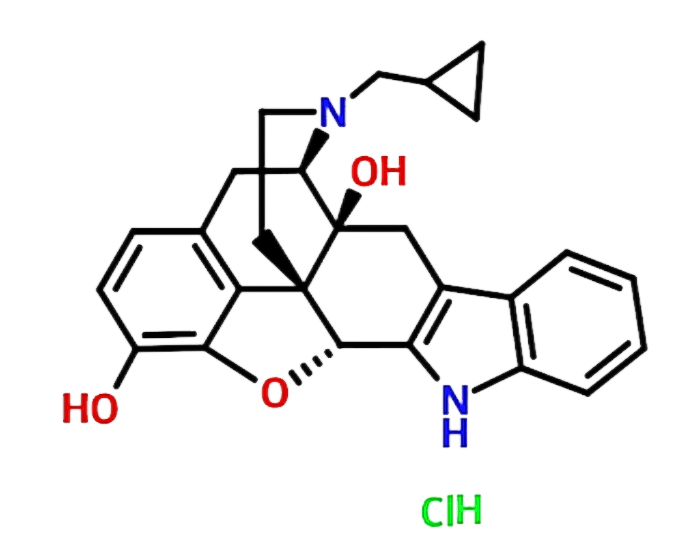


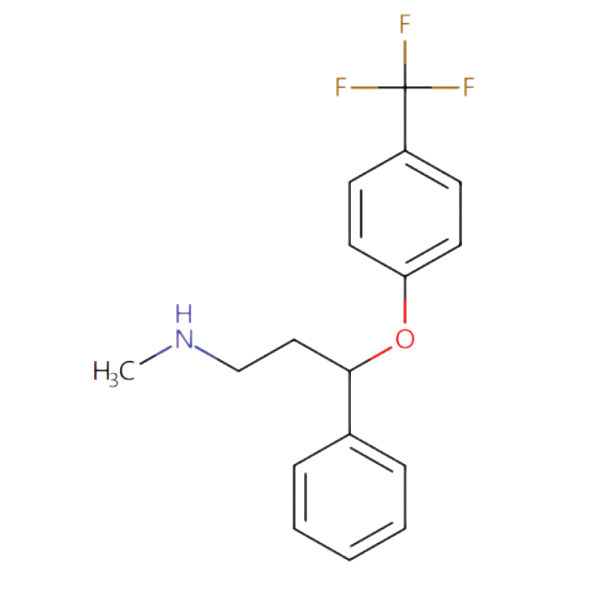

Supplement: Supplementary 4 — Supplementary Figure 4: 2D structure of the candidate compounds. [file 3677532.f4.docx]
