# Supplementary material for: Bioinformatic Exploration of Hub Genes and Potential Therapeutic Drugs for Endothelial Dysfunction in Hypoxic Pulmonary Hypertension
Source: Comput Math Methods Med. 2022 Nov 28;2022:3677532. doi: 10.1155/2022/3677532 (PMC9723419; doi:10.1155/2022/3677532)
Supplement: Supplementary 5 — Supplementary Table 1: characteristics of the individual dataset. [file 3677532.f5.docx]

**Supplementary Table 1** Characteristics of the individual dataset

| **GEO ID** | **Platform** | **Cell type** | **Sample size** | **Country** | **Time** |
| --- | --- | --- | --- | --- | --- |
| GSE11341 | GPL96 (Affymetrix Human Genome U133A Array) | cardiac microvascular endothelial cells | 3 vs. 9 | Ireland | 2018 |
| GSE160255 | GPL23159 (Affymetrix Clariom S Assay, Human (Includes Pico Assay) | pulmonary artery endothelial cells | 3 vs. 3 | United States | 2021 |
