# Supplementary material for: Bioinformatic Exploration of Hub Genes and Potential Therapeutic Drugs for Endothelial Dysfunction in Hypoxic Pulmonary Hypertension
Source: Comput Math Methods Med. 2022 Nov 28;2022:3677532. doi: 10.1155/2022/3677532 (PMC9723419; doi:10.1155/2022/3677532)
Supplement: Supplementary 6 — Supplementary Table 2: the top 10 hub genes of 7 algorithms in CytoHubba. [file 3677532.f6.docx]

**Supplementary Table 2** The top 10 hub genes of 7 Algorithms in cytohubba

| Algorithm | Gene |
| --- | --- |
| MNC | ACVR1B; WIF1; NTRK1; LOX; VEGFA; CDC25A; GRIA1; MCM4; FGF9; HMOX1 |
| Degree | DNM2; HMOX1; LOX; GRIA1; NTRK1; ACVR1B; MAPK4; CDC25A; VEGFA; HPGD |
| EPC | HMOX1; FGF9; VEGFA; CD24; NTRK1; CDC25A; LOX; MAPK4; WIF1; ACVR1B |
| Betweeness | INHBA; VEGFA; CDC25A; FGF9; LEF1; WIF1; LOX; NTRK1; PTGER4; CD24 |
| Closeness | NTRK1; LOX; VEGFA; CDC25A; DNM2; PTGER4; PTPN22; MAPK4; GRIA1; ACVR1B |
| Radiality | NTRK1; LOX; VEGFA; CDC25A; DNM2; PTGER4; ACVR1B; GRIA1; MAPK4; PTPN22 |
| Stress | GRIA1; NTRK1; LOX; VEGFA; CDC25A; PTPN22; TF; MAPK4; HPGD; DNM2 |
